# From linked open data to molecular interaction: studying selectivity trends for ligands of the human serotonin and dopamine transporter†
†The authors declare no competing interests.
‡
‡Electronic supplementary information (ESI) available. See DOI: 10.1039/c6md00207b



**DOI:** 10.1039/c6md00207b

**Published:** 2016-07-22

**Authors:** Barbara Zdrazil, Eva Hellsberg, Michael Viereck, Gerhard F. Ecker

**Affiliations:** a Department of Pharmaceutical Chemistry , Pharmacoinformatics Research Group , University of Vienna , Althanstraße 14 , A-1090 , Austria . Email: barbara.zdrazil@univie.ac.at ; Fax: +43 1 4277 55113 ; Tel: +43 1 4277 55110

## Abstract

Retrieval of congeneric and consistent SAR data sets for protein targets of interest is still a laborious task to do if no appropriate in-house data set is available. However, combining integrated open data sources (such as the Open PHACTS Discovery Platform) with workflow tools now offers the possibility of querying across multiple domains and tailoring the search to the given research question. Starting from two phylogenetically related protein targets of interest (the human serotonin and dopamine transporters), the whole chemical compound space was explored by implementing a scaffold-based clustering of compounds possessing biological measurements for both targets. In addition, potential hERG blocking liabilities were included. The workflow allowed studying the selectivity trends of scaffold series, identifying potentially harmful compound series, and performing SAR, docking studies and molecular dynamics (MD) simulations for a consistent data set of 56 cathinones. This delivered useful insights into driving determinants for hDAT selectivity over hSERT. With respect to the scaffold-based analyses it should be noted that the cathinone data set could be retrieved only when Murcko scaffold analyses were combined with similarity searches such as a common substructure search.

## Abstract 2

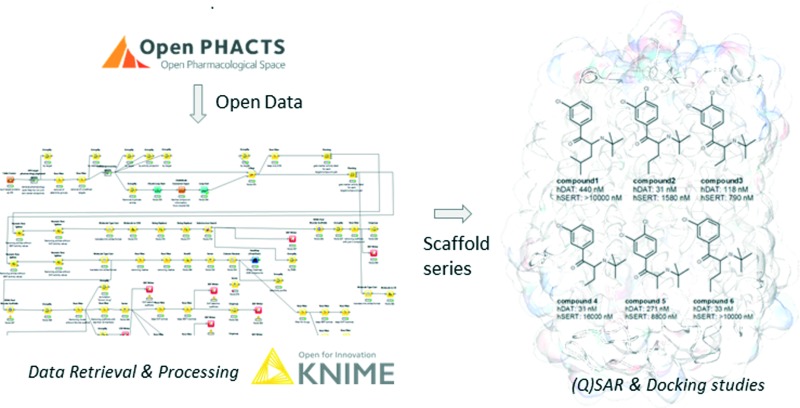
Retrieval of consistent SAR data sets is a challenging task. Combining integrated open data sources with workflow tools allows studying selectivity trends of compound series.

## Introduction

With the public availability of large data sources such as ChEMBL^1^ and the Open PHACTS Discovery Platform,^2^ retrieval of data sets for certain protein targets of interest measured under consistent assay conditions is no longer a time-consuming process. Especially the use of workflow engines such as KNIME^3^ or Pipeline Pilot^4^ allows the submission of complex queries and enables simultaneous searching for several targets. This has recently been demonstrated for two ABC transporters, where the use of the Open PHACTS API delivered useful data sets for subsequent classification models.^5^ However, extracting data sets suitable for QSAR studies still remains a challenge due to the special requirements needed for performing quantitative data analyses.^6^ These include, among others, the demand for a homologous series of compounds measured under comparable assay conditions. In order to assess the capabilities of the Open PHACTS Discovery Platform for providing such data sets, two representatives of the solute carrier (SLC) family were selected for a proof-of-concept study.

Solute carriers represent the largest group of transporters in the human genome, containing more than 400 representatives.^7^ This includes several prominent and important drug targets, such as the human sodium-dependent serotonin transporter (serotonin transporter or hSERT) and the human sodium-dependent dopamine transporter (dopamine transporter or hDAT).

These transporters belong to the solute carrier 6 (SLC6) gene family, also referred to as the neurotransmitter sodium symporter (NSS) family or as Na^+^/Cl^–^-dependent transporters.

Numerous compound classes have been identified to interact with these transporters, and they are used in therapeutic settings or abused as illicit drugs.^8,9^ The therapeutic spectrum includes *inter alia* tricyclic antidepressants, selective serotonin reuptake inhibitors (SSRIs) and stimulant agents, whereas MDMA (3,4-methylendioxymethamphetamine), cocaine and the methamphetamines are prominent representatives of the abusive range, though admittedly the borders blur between treatment and malpractice.^10,11^ The activation of dopamine receptors in certain brain regions plays an important role in causing addiction. Addictive drugs elevate the dopamine levels in these regions, making it more available to the receptors – for example by interfering with the reuptake by the dopamine transporter.^12^ However, drugs interacting with hDAT in a therapeutic setting do not cause abusive effects. Serotonin and its pathways are not involved in the reward system and therefore cause no addiction. The potential to cause an addictive tendency or not is an important piece of information to keep in mind for a meaningful selectivity profiling.

A quite prominent group of illicit drugs is the class of cathinones. They are sold as bath salts, research chemicals or plant food to avoid detection by authorities.^13^ Following the report of European drug monitoring in 2015, 450 new psychoactive substances were traced by the European warning system (EWS) with 31 new synthetic cathinones amongst them.^14^ Out of these representatives, mephedrone and methylenedioxypyrovalerone are among the most prevalent cathinones.^15,16^ Structure–activity relationship (SAR) studies as well as docking of selected cathinones into protein homology models of hDAT and hSERT revealed the first insights into the molecular basis of transporter selectivity.^15,17,18^

Following the aim of this study, we explored the whole chemical space of hSERT/hDAT interacting compounds in the open domain and analysed the results with respect to scaffolds appearing to be selective for either hSERT or hDAT. Subsequently, a data set of 56 cathinone analogues measured on both transporters was extracted and used for ligand- and structure-based modelling studies. This led to further insights into the molecular features that drive transporter selectivity.

## Results and discussion

### Data retrieval and analyses

**Fig. 1:**
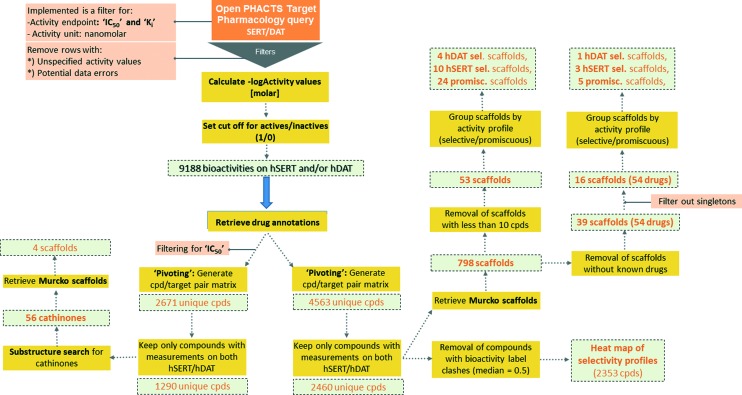
Schematic depiction of the KNIME workflow for data retrieval, filtering, processing and analyses.

Semantically integrated data sources such as the Open PHACTS Discovery Platform^2^ are a powerful tool to conduct complex queries in the life sciences domain.^19^ In analogy to a recent study on a set of ABC transporters,^5^ chemical compound bioactivity data for human SERT and DAT were retrieved from the Open PHACTS Discovery Platform by utilizing a KNIME workflow (Fig. 1).^3^

From the beginning, data retrieval was restricted to the activity endpoints IC_50_, and *K*_i_. After filtering for ‘single protein’ targets and preprocessing, 5405 bioactivities for hSERT, and 3783 bioactivities for hDAT remained (9188 data points in total: 4698 IC_50_, 4490 *K*_i_ values). By creating an overlap matrix *via* mapping ChEMBL compound IDs, 4563 unique compounds were retained (2671 for IC_50_ only).

Being aware that mixing activity data from different assays with endpoint IC_50_ introduces noise/uncertainty to the analysis, an investigation on intra- and intervariability in different pIC_50_ and p*K*_i_ measurements for hSERT and hDAT was performed. Correlating pIC_50_ to p*K*_i_ values (intervariabilities) from duplicate measurements led to an *R* ^2^ of 0.62 for hSERT (385 compounds) and 0.75 for hDAT (360 compounds). These values are in the same range as intravariabilities of p*K*i and pIC_50_ values if the maximum and minimum values of multiple measurements are correlated (p*K*_i_ correlation: *R* ^2^ = 0.74 for hSERT, *R* ^2^ = 0.69 for hDAT; pIC_50_ correlation: *R* ^2^ = 0.61 for hSERT, *R* ^2^ = 0.76 for hDAT). Thus, we can assume that the size of error gained from mixing different assay outcomes in our setup is in the same range as the error introduced by multiple measurements of the same compound–target pair.

For a global scaffold analysis of the hSERT/hDAT chemical space present in the Open PHACTS Discovery Platform, we thus kept both bioactivity endpoints (IC_50_ and *K*_i_) but filtered out entries without measurements on both transporters. This led to a total number of 2460 unique compounds with median and mean activity labels assigned.

Cut-offs for separating actives from inactives (and assigning the respective label 1/0) were tailored to the specific protein (hSERT/hDAT) and activity endpoint (*K*_i_/IC_50_). Known drugs included in the data sets were ranked according to their bioactivity values for that target (for IC_50_ and *K*_i_ values separately). The drug with the lowest bioactivity which is still recognized as being pharmacologically active on hSERT or hDAT in DrugBank^20^ (Version 4.5) was used as a reference and its bioactivity served as a tailored cut-off for that target and endpoint. Thus, in the case of hSERT, sibutramine (*K*_i_ = 1.11 μM; IC_50_ = 2.09 μM), and in the case of hDAT, modafinil (*K*_i_ = 1.46 μM; IC_50_ = 1.83 μM), were selected as a reference.

**Fig. 2:**
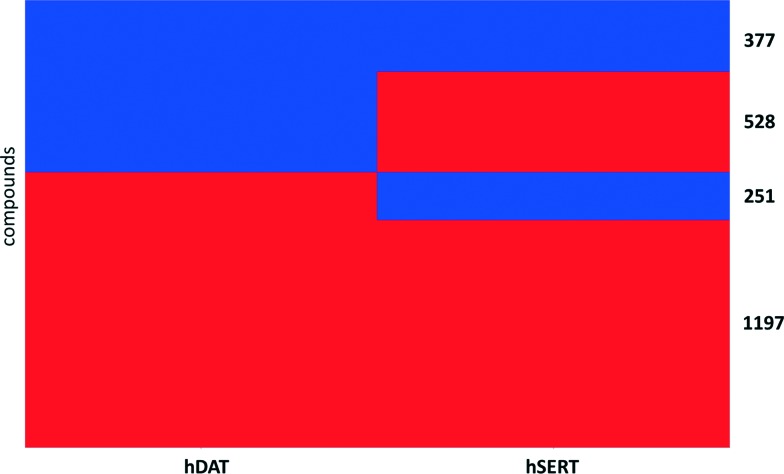
Heat map reflecting the selectivity profile of 2353 unique compounds with bioactivity measurements (IC_50_ and *K*_i_) for human SERT and DAT in binary representation. Red bars, active; blue bars, inactive.

For the purpose of showing the selectivity profiles of the 2460 unique compounds in a heat map representation, instances with a median activity label of 0.5 (meaning that they were found active/inactive in different measurements or assays) were removed from the data set, leading to a matrix of 2353 compounds (Fig. 2). As can be seen from the heat map, more than half of the compounds are active on both hSERT and hDAT (1197) in the low μmolar or submicromolar range, and the smallest proportion of compounds (251) is the one showing selectivity for hDAT over hSERT, whereas hSERT selectives are clearly overrepresented (528 compounds). This might be due to the fact that SSRIs (selective serotonin reuptake inhibitors) represent a prominent class of antidepressant drugs. Further evidence for this selectivity bias can be retrieved by analysing the selectivity profiles of drugs within this heat map: eighteen hSERT-selective *versus* eight hDAT-selective marketed drugs are present, with a large portion of antidepressants in the hSERT-selective cluster.

Subsequently, Bemis–Murcko scaffolds for the 2460 unique compounds were computed as part of the KNIME workflow. Strikingly, clustering the data set by scaffolds led to a total of 798 unique scaffolds with 745 of those scaffold clusters comprising less than ten member compounds. The high number of different scaffolds most probably is due to the fact that the generation of scaffolds according to Bemis and Murcko distinguishes between different stereoisomers. Furthermore, it is not possible to treat certain heteroatoms as optional. Thus, a medicinal chemistry perspective is certainly needed for drawing conclusions from such clustering.

**Fig. 3:**
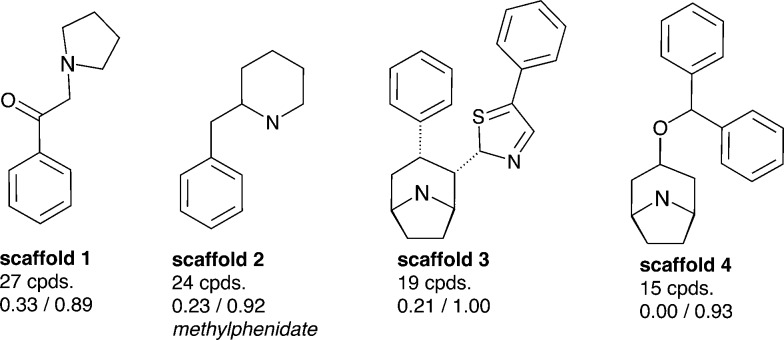
Four rather hDAT-selective scaffold clusters (considering IC_50_ and *K*_i_) with counts of unique compounds within this cluster and mean values of activity labels of their member compounds: lower left (hSERT), lower right (hDAT); representative drugs contained in these scaffold clusters are mentioned if available; molecules are depicted without hydrogens.

**Fig. 4:**
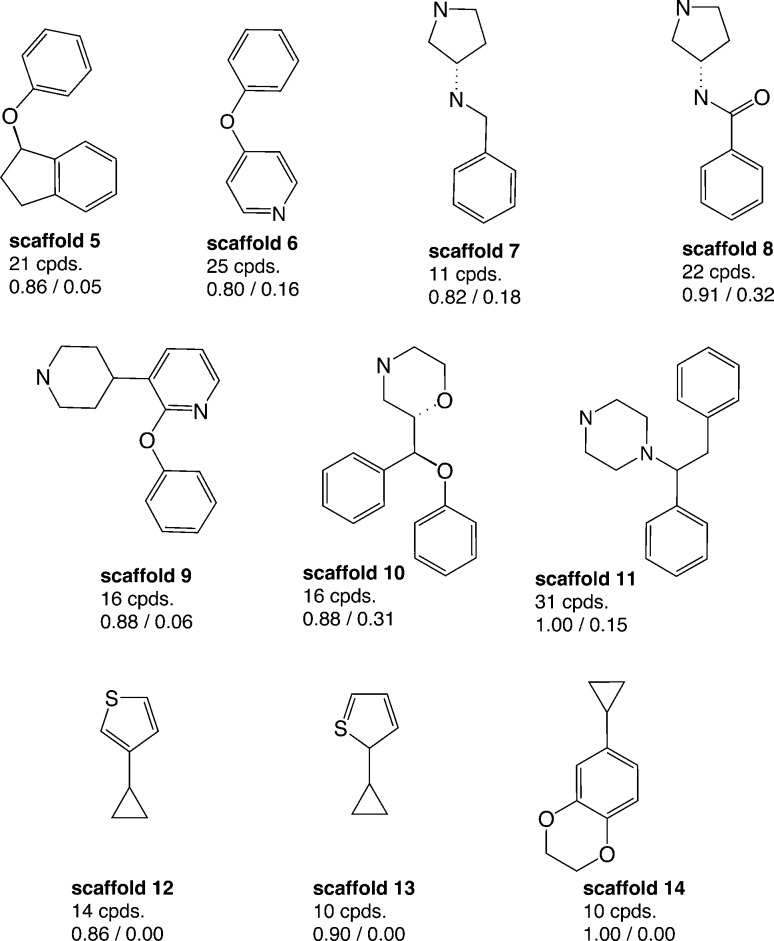
Ten rather hSERT-selective scaffold clusters (considering IC_50_ and *K*_i_) with counts of unique compounds within this cluster and mean values of activity labels of their member compounds: lower left (hSERT), lower right (hDAT); molecules are depicted without hydrogens.

Aiming to identify hSERT/hDAT-selective scaffolds *versus* promiscuous ones among the higher populated 53 scaffolds (with at least ten member compounds), the mean values (between 0 and 1) of activity labels (0/1) of respective member compounds were analysed. A mean value below or equal to 0.4 points to a trend towards inactivity within the scaffold series, a value above or equal to 0.6 towards activity, whereas mean activity labels closer to 0 for inactives, or *vice versa* closer to 1 for actives, are pointing towards more pronounced trends within a scaffold cluster. This led to four rather hDAT-selective scaffolds, 10 rather hSERT-selective scaffolds, and 24 scaffolds with a pronounced activity on both transporters (Fig. 3, 4 and S1, ESI‡). The remaining 15 scaffolds are either inactive on both hSERT and hDAT (5 scaffolds), or no clear activity trend can be deduced among the member compounds (displaying a mean value of assigned activity labels for hSERT or hDAT of around 0.5; 10 scaffolds).

Regarding the final selection of hSERT- and hDAT-selective scaffold series (Fig. 3 and 4), some scaffolds appear structurally very similar. For instance, the only difference between scaffolds 12 and 13 (Fig. 4) is the position of the sulfur atom in the thiophene ring. Also, mean bioactivity labels of these two scaffolds reflect equivalent selectivity trends (Fig. 4), which suggests that also SAR trends might be coherent. It needs a medicinal chemist's experience to inspect such similar scaffold clusters in more detail and decide whether the compound series could be merged due to the existence of a common substructure required for the interaction with the target protein.

Surprisingly, although many more rather hSERT-selective scaffolds *versus* hDAT-selective scaffolds were found (4 *vs.* 10 scaffolds), the reason for this imbalance is not the existence of antidepressants within these scaffold clusters, as was the case when all individual compounds in the heat map were examined (Fig. 2). On the contrary, we could not find any marketed drug within the 10 hSERT-selective scaffold series (Fig. 4). For hDAT, at least one scaffold series contains a drug (methylphenidate in scaffold 2, Fig. 3).

### Where are the drugs?

**Fig. 5:**
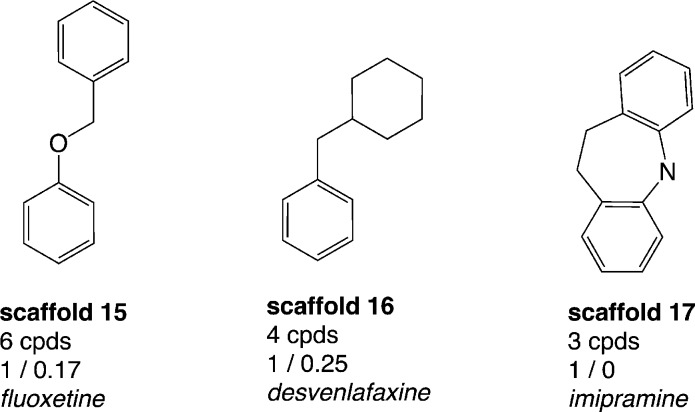
Drug-containing hSERT-selective scaffolds (considering IC_50_ and *K*_i_) with counts of unique compounds within this cluster, and mean values of activity labels of their member compounds: lower left (hSERT), lower right (hDAT); representative drugs contained in these scaffold clusters are mentioned; molecules are depicted without hydrogens.

Thus, a thorough analysis of the distribution of drugs within the data set was performed in order to additionally identify scaffolds which already proved to be important from a drug-discovery perspective. The total number of drugs in the whole data set with annotations for both hSERT and hDAT (2460 compounds) is 54 (sd file available in the ESI,‡ File S2). These drugs can be assigned to 39 (out of 798) scaffolds. Strikingly, 23 out of these 39 drug-containing scaffold clusters appear as singletons in our analyses, which suggests that along with these drugs there were no proper SAR series published. However, 18 out of these 23 scaffolds are composed of at least 3 rings and in general are quite complex. Still, some of the scaffolds are structurally very similar (*e.g.* ketoconazole and terconazole), which indicates that the scaffold extraction algorithm does not allow identification of the SAR series. Grouping the remaining 16 drug-containing scaffolds (with more than one member compound) by rather hSERT-selective, hDAT-selective and promiscuous scaffolds, we again found a larger proportion of hSERT-selective scaffolds over hDAT (3 *vs.* 1 scaffold), with a greater number of drugs in total for hSERT selectives (10 drugs *versus* 1). However, hSERT-selective scaffolds appear rather sparsely populated with less than ten unique compounds per cluster (Fig. 5), whereas the single drug-containing hDAT-selective scaffold 2-benzylpiperidine (scaffold 2 in Fig. 3) is composed of 24 compounds with just one annotated drug (methylphenidate). Such compound series, comprising a common scaffold, a clear selectivity trend, and being populated by at least one drug, are ideal starting points for further SAR studies (see ‘The cathinone use case’ section). In contrast, the rather hSERT-selective drug-containing scaffolds (scaffolds 15–17, Fig. 5) comprise a very low number of respective member compounds (3–6) with 50–100% of their compounds being annotated drugs. Within these clusters, the tricyclic antidepressants (TCAs) imipramine, clomipramine, and desipramine are located, as well as antidepressants of the selective serotonin reuptake inhibitor (SSRI) class (*e.g.* fluoxetine) and of the serotonin–norepinephrine reuptake inhibitor (SNRI) class (*e.g.* venlafaxine). It seems rather surprising that these well-known drug classes would not show up in congeneric SAR series (with at least a few member compounds), which reflects the communities' synthetic efforts and interest in a certain drug class. We therefore performed a substructure search, looking for the four drug-containing SERT-selective scaffold types. For this search, scaffolds from Fig. 5 were further refined if a common substructure bigger than the Murcko ring system was contained within the scaffold series (*e.g.* by adding an aminoethyl side chain in the case of scaffold 15 and a dimethylaminomethyl side chain in the case of scaffold 16). As expected, additional compounds could be retrieved from the hSERT/hDAT data set. Surprisingly, for scaffold 17 (imipramine-type scaffold) only one additional compound could be found showing moderate activity on hSERT. These tricyclic antidepressants and derivatives were not in the focus of synthetic efforts within the last decade and therefore do not show up with the same prevalence in the MedChem literature extracted from ChEMBL as is the case for SSRI- and SNRI-like compounds. A survey on the prevalence of imipramine- and fluoxetine-reporting publications in ChEMBL revealed that fluoxetine was reported 3 times more often since 1993. ChEMBL basically covers publications reporting on SAR series (where imipramine and fluoxetine bioactivities would have been reported also). Therefore, these numbers are somewhat representative of the amount of analogues in these publications. An equivalent search in SciFinder^21^ with publications going back to the 1980s supports these findings, showing a ten-year shift in peaks of fluoxetine- *versus* imipramine-containing publications (see Fig. S3, ESI‡). However, also regarding SSRI- and SNRI-like compounds, these analogues show up in diverse Murcko scaffold clusters within our data set depending on the presence of additional rings. For scaffold 16, five additional compounds could be retrieved. For scaffold 15, even 170 extra compounds appeared with various different Murcko scaffolds still possessing the same common substructure. Moreover, one should not forget that these substructure searches were performed on the data set with measurements for both transporters only. Repeating the same analysis on the bigger data set before the removal of compounds with missing measurements for one of the two proteins, even more compound analogues were detected (data not shown).

Finally, inspecting the five promiscuous drug-containing scaffold series (possessing mean activity labels ≥0.6 for both hSERT and hDAT), one additional marketed antidepressant belonging to the class of SSRIs was detected: sertraline. It belongs to a rather big scaffold cluster of 48 compounds (see Fig. S1, ESI‡), and although potent activity on hDAT has been reported for the whole compound series, according to DrugBank^20^ the pharmacological action on hDAT is ‘unknown’. The remaining four scaffolds include, *e.g.* the drugs sibutramine and mazindol, with pharmacological effects and side effects similar to those of amphetamines. Both are classified as anorectics; however, mazindol is not marketed for use in the treatment of obesity and sibutramine was withdrawn from the market in 2010 due to severe cardiovascular (CV) side effects. Recently, these sibutramine-induced CV adverse events have been attributed to hERG (human ether-à-go-go-related gene) channel inhibition.^22^ HERG encodes channels responsible for the cardiac rapid delayed rectifier potassium current. Blocking hERG by small molecules and drugs is related to QT interval prolongation and cardiac arrhythmia (torsades de pointes, TdP). Consequently, in drug-discovery projects compounds are commonly screened in the early phases against hERG in order to avoid such potential side effects.^23^

### Flagging blockers of the hERG potassium channel

In this context, it appears interesting to investigate the potential of extracted scaffold series to inhibit the hERG channel by flagging single compounds within a series if an inhibitory effect was measured (<10 μM). Although it is hard to assess if the ability to interact with hERG is rather induced by a specific scaffold or by a certain side chain (or a combination of both), in some cases such serial trends have been reported, *e.g.* for some tricyclic antidepressants.^24^

In a separate workflow, we therefore included pharmacology data on hERG for the 2460 unique compounds (with both hSERT and hDAT measurements). For hDAT selective scaffold series we did not get any alert on hERG inhibition, within hSERT selective scaffold series, however, some hERG blockers could be identified (9 out of 13 compounds belonging to the scaffold series 15–17, Fig. 5). This is in so far alarming as many of the approved drugs are also hERG inhibitors. Out of 54 drugs with measurements for both hSERT and hDAT, we identified 19 drugs with a potential liability due to hERG blockage; six out of these are marketed antidepressants. In addition, it was reported previously that a 30-fold safety margin between the effective therapeutic free plasma concentration and hERG IC_50_ should be met in order to prevent QT interval prolongation.^25,26^ These studies revealed that indeed some marketed antidepressants (*e.g.* amitryptiline, citalopram, imipramine, fluoxetine) might be associated with QT interval prolongation and TdP.

Using the information on hERG blocking liabilities provided by the workflow, potentially harmful compound series can be identified at an early stage in the drug discovery pipeline if data on hERG inhibition is available in the open domain. For the assessment of the risk of dTP, however, an additional literature survey or *in vitro*/*in vivo* studies are needed in individual cases.

Although it is not in the focus of the underlying investigation to study other potential off-target effects (*e.g.* interaction with GPCRs, ABC transporters, *etc.*), the workflow provides the flexibility to include any target pharmacology desired in the context of the use case of interest.

### The cathinone use case

Our studies point to the fact that Murcko scaffold analyses have to be always interpreted with caution, as certain structurally very similar scaffolds (possessing a common substructure) could fall into different scaffold clusters and would therefore sometimes be filtered out if strict counts of member compounds serve as filtering criteria. This is, *e.g.*, the case for the hDAT-selective scaffold 1 (Fig. 3), which clearly relates to the group of cathinones, a subclass of amphetamines currently comprising popular illicit drugs with a rising trend of consumption.

**Fig. 6:**
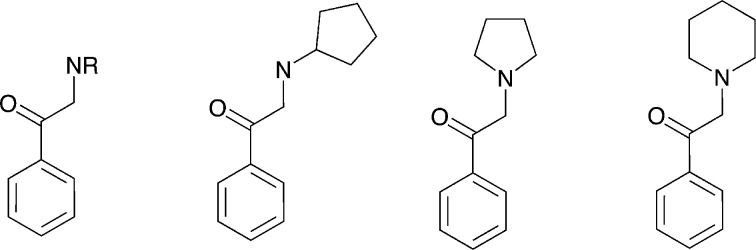
Four different scaffold types in the cathinone data set in Murcko representation (except for the very left scaffold which would be benzene in Murcko representation); hydrogens are not depicted.

Filtering the initial data set retrieved by the KNIME workflow for IC_50_ bioactivity endpoints created an overlap matrix of 2671 unique compounds, with 1290 compounds having measurements for both transporters. A substructure search with the cathinone structure (= benzoylethanamine) as input led to a final cathinone data set of 56 unique compounds (an sd file of the cathinone data set can be found in the ESI,‡ File S4), reported essentially in three different publications.^27–29^ Just two compounds, pyrovalerone (CHEMBL201960) and bupropion (CHEMBL894), have been reported in other additional articles.^30–33^ Having a closer look at these 56 cathinones, one essentially captures four different scaffold types (Fig. 6). They are all showing the cathinone substructure, either possessing an aliphatic side chain (falling into the large benzene cluster), a cyclopentane substituent on the amine group, or having the amine as part of a five- or six-membered saturated ring (pyrrolidine or piperidine). This again outlines the drawback of a scaffold definition based primarily on the number of rings, as obviously all four compound series belong to what a medicinal chemist would label as a cathinone-like structure.

### SAR analysis

Although, activity values for the 56 cathinones were retrieved from three different publications,^27–29^ assay parameters were identical. Therefore, these IC_50_ measurements could be combined into one data set suitable for SAR studies. As the publications were mainly aiming at investigating hDAT, around 50% of the compounds just show >10 μM or >100 μM for hSERT. Thus, quantitative statements linking structural features to transporter selectivity need to be taken cautiously. The main structural variations comprise the substituent of the nitrogen atom, the substituents at the aromatic ring, as well as some modifications at the C_α_ to the carbonyl group. As already outlined in a previous study, a pyrrolidine ring at the nitrogen atom strongly favours hDAT selectivity.^15^ Compounds with *t*-butyl and piperidine substituents show analogous behavior. Also the substituent in the α-position to the carbonyl group seems to contribute, with larger groups fostering hDAT selectivity.^34^ However, in most of the cases transporter selectivity is achieved by rendering the compounds less active or inactive at hSERT rather than improving hDAT binding. To further analyse this, we performed multiple linear regression on this data set with hDAT pIC_50_ values as well as selectivity as dependent variables. As the main purpose of this study was to get further evidence on SAR trends observed, we just used a very limited set of descriptors. These comprise the overall van der Waals volume (vdw-vol), the partition coefficient (log *P* (o/w)) and molar refractivity (mr) of the compounds, the van der Waals volume of the substituent at the C_α_-atom to the carbonyl group (vdw-vol-C_α_), the van der Waals volume of the substituent at the nitrogen atom (vdw-vol-N) as well as substituent constants for the substituents at the aromatic ring (π-arom, mr-arom, *σ*_m_ and *σ*_p_) and indicator variables for meta (*I*_m_) and para substitutions (*I*_p_). The analysis was performed using StatPlus for Mac, starting with all variables and performing a backward descriptor selection until all regression coefficients showed 95% confidence. The following equation was obtained for hDAT pIC_50_ (eqn. (1)):

1 hDAT pIC_50_ = 7.01 – 0.63 log *P* + 0.03 vdw-vol-C_α_ + 0.91 π-arom*n* = 51, *r*^2^ = 0.56

Using the log(IC_50__SERT/IC_50__DAT) as the dependent variable (corresponding to log selectivity), the qualitative trends discussed above could be further strengthened (eqn. (2)), whereby para-σ was borderline with respect to significance:

2 Log selectivity = 30.3 – 7.05 mr + 0.12 vdw-vol-N + 0.15 vdw-vol-C_α_ + 0.69 mr-arom – 0.97*σ*_p_*n* = 25, *r*^2^ = 0.56

Both equations point towards a significant influence of the substituent at the C_α_-atom to the carbonyl group on the hDAT activity as well as on hDAT over hSERT selectivity of the compounds. This is *e.g.* exemplified by compound CHEMBL202409 (File S4, ESI‡), which has an isobutyl moiety in this position and shows a 345-fold selectivity for hDAT.

### Molecular docking

**Fig. 7:**
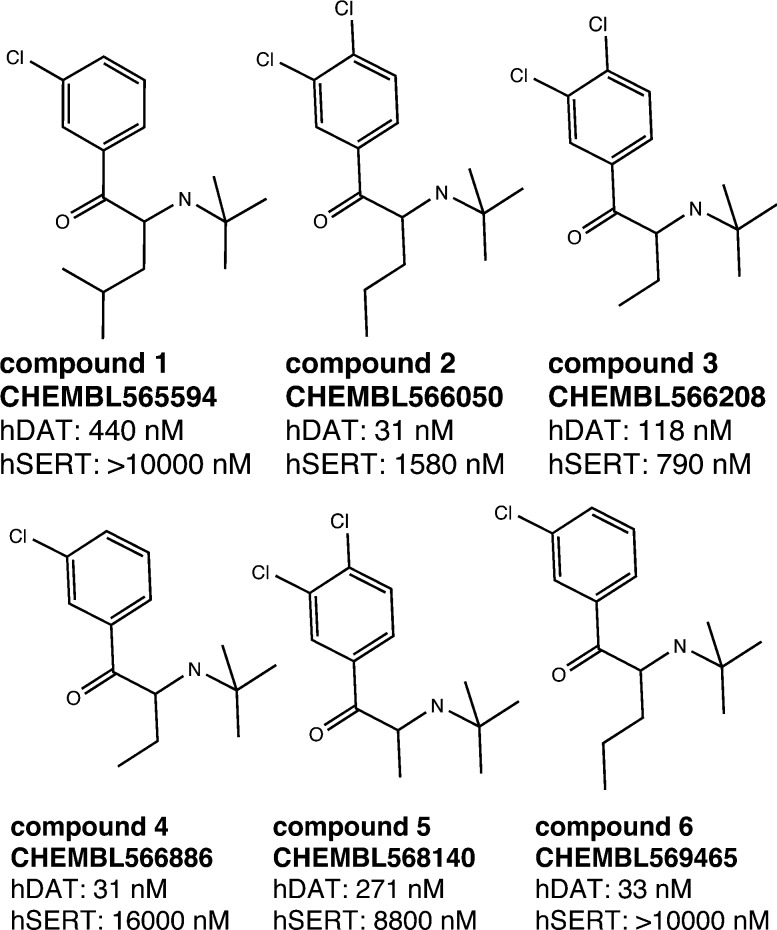
Selected compounds for the docking study. The activity values represent the measured IC_50_ results from the literature.^27–29^ If multiple measurements were available, we considered the lowest IC_50_.

As outlined above, both SAR studies as well as multiple linear regression analysis point towards a role of the C_α_-substituent for hDAT over hSERT selectivity of cathinones. We thus selected a set of compounds, which show variation in this position, for docking studies into protein homology models of the two transporters.^15^ As seen in Fig. 7, all six compounds are rather inactive at hSERT (considering a cut-off of 1–2 μM), while at hDAT they are all active (showing bioactivities in the range of 31 nM to 440 nM). While previous modelling studies focused on the substitutions at the aromatic ring and at the cationic nitrogen,^15,18,35,36^ the compounds chosen in this study are supposed to provide information about the role of the C_α_-substituent.

In order to derive potential structure-based hypotheses for hDAT over hSERT selectivity of cathinones, we docked these six cathinones (Fig. 7) into homology models of hDAT and hSERT. The central binding site of the biogenic monoamine transporters is divided into the three subsites A, B and C.^37^

**Fig. 8:**
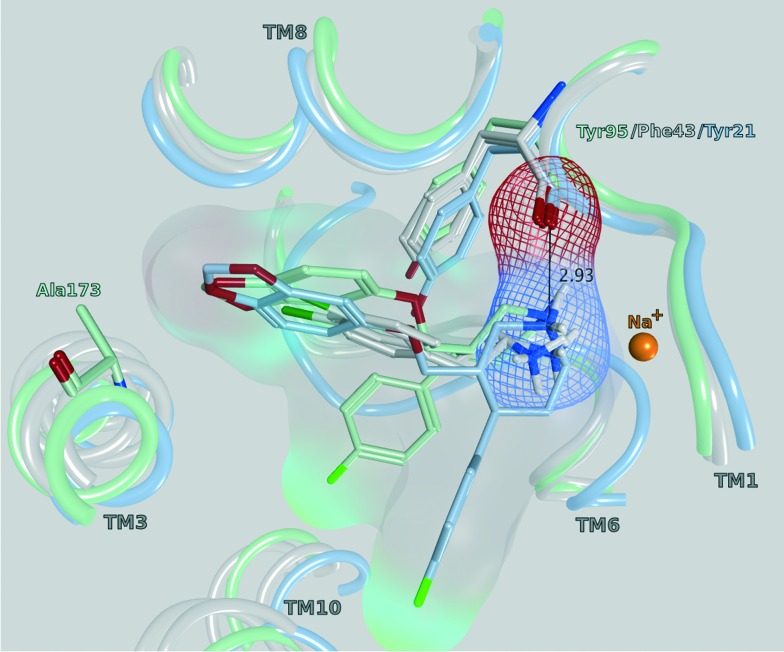
Central binding site (side view) of PDB 4XP9 (grey, dDAT with d-amphetamine), ; 4XPA (grey, dDAT with 3,4-dichlorophenethylamine), ; 4MM4 (light blue, LeuBAT with paroxetine) and ; 5I6X (mint, hSERT with paroxetine), pointing out the vicinity of the cationic nitrogen to the carbonyl oxygen of Tyr95 (hSERT), Phe43 (dDAT) and Tyr21 (LeuBAT), respectively. The marked distance of 2.93 Å is measured in ; 4MM4.

All of the monoamine transporter crystal structures in the PDB (14 of dDAT, 12 of LeuBAT and 5 of hSERT) comprise the same orientations of their co-crystallized ligands: the cationic nitrogens reaching into subsite A and the aromatic moieties pointing towards subsite B or C of the central binding cavity. Further, the influence of Phe76 in hDAT and Tyr95 in hSERT on an appropriate transport rate is well known from mutational studies.^10,38–40^ In Fig. 8, a selection of four PDB structures (; 4XP9, ; 4XPA, ; 5I6X, ; 4MM4) is depicted in order to demonstrate this common orientation and the vicinity to the mentioned amino acids. The structures were selected in a way (a) as to represent each crystallized monoamine transporter and (b) because they have been co-crystallized with ligands. d-Amphetamine and 3,4-dichlorophenethylamine were chosen due to the structural similarity to the cathinone series under investigation. Paroxetine was selected once because the co-crystallized ligand of ; 4MM4 was used for the homology model of hSERT, and a second time in ; 5IX6 because it has the highest resolution in the very recently released hSERT crystals.^41^ A previous docking study by Sakloth *et al.* ^36^ shows the same orientation and vicinities of p-substituted cathinones. Resulting from these observations, the cationic nitrogen in the cathinones was restrained to be placed within 2–4 Å to the backbone of the carbonyl oxygen of Phe76 in hDAT and Tyr95 in hSERT in the actual docking study, as published already by Saha *et al.* ^15^

Following our common scaffold clustering method,^42–44^ we obtained five different clusters in hDAT composed of a total of 103 poses. The co-crystallized structures of LeuBAT^45^ and dDAT^46^ show that the aromatic moiety of the ligands is primarily placed in the B-site and to a much lesser extent also in the C-site. Two of the dDAT structures are co-crystallized with methamphetamine (4XP6) and d-amphetamine (; 4XP9), which are structurally similar to the cathinones. Both compounds show a methyl group in the C_α_-position which points to the center of the binding site. Based on these experimental findings, we selected the clusters 1 (38 poses) and 2 (34 poses) (Fig. S5, ESI‡) for further analysis, as in these clusters the aromatic moieties protrude into the B- and C-site, respectively (Fig. 8). Additionally, these clusters contain the majority of the retrieved poses. Clusters 3 (13 poses) and 4 (9 poses) are remarkably smaller and there is no experimental evidence for these placements. Cluster 5 (9 poses) is similar to cluster 2 with the aromatic moiety pointing into the C-site, but the orientation of the carbonyl oxygen and the C_α_-substituent is diametrically opposed.

**Fig. 9:**
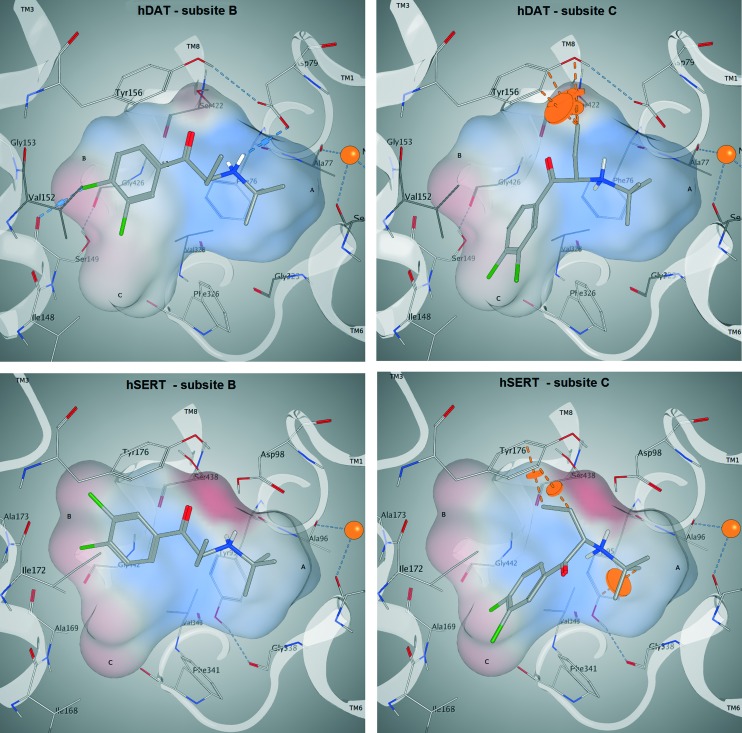
Docking poses of compound 3 (CHEMBL566208) in hDAT (top) and hSERT (bottom). In both proteins, the aromatic moiety reaches into the B-site (left; corresponds to cluster 1) and in the C-site (right; corresponds to cluster 2). In the C-site, steric clashes with the C_α_ substituent are visible (dashed orange lines). An H-bond is only found in hDAT if the aromatic moiety reaches into the B-site (top left, dashed blue lines).

In cluster 1 (Fig. S5, ESI‡), all six compounds are present, with the aromatic moieties reaching into the B-site and the cationic nitrogens being close to the carbonyl oxygen of Phe76. However, the latter is due to the constraint set during pose generation (see Methods). The majority of C_α_ substituents points to the center of the binding site, where the most space is provided (Fig. 9, upper left). However, in a small number of poses the C_α_ substituent is turned towards Asp79, Tyr156 and Val152, which might lead to a spatial hindrance as the relevant six poses are showing steric clashes with these amino acids. Additionally, in 19 poses an H-bond interaction between the cationic nitrogen and Asp98 could be observed.

In cluster 2 (Fig. S5, ESI‡), the aromatic moieties are located in the C-site and the majority of the C_α_ substituents are facing towards Asp79, Val152, Tyr156 and Ser422 (Fig. 9, upper right), which leads to 19 clashing poses (of 34 in total). Further, in cluster 5, which shows the same orientation of the aromatic ring, the C_α_ substituents have similar problems with Tyr156 and Ser422, as eight out of nine poses show steric clashes.

In hSERT, the results look quite similar (Fig. 9, bottom left and right), which might be expected considering the high sequence identity between the two proteins in the binding site. Here we obtained 65 different poses in four separate clusters. The pattern of distribution is highly comparable to the one observed in hDAT: cluster 1 (Fig. S6, ESI‡) includes 31 poses with the aromatic moieties reaching into the B-site. However, in contrast to hDAT, no H-bonds with the cationic nitrogen are found. Cluster 2 (Fig. S6, ESI‡) comprises 22 poses with the aromatic ring positioned in the C-site and a high number of steric clashes with Asp98, Tyr176 and Ser438 due to the C_α_ substituents. Cluster 3 (9 poses) and 4 (3 poses) are remarkably smaller and located like their counterparts in hDAT.

Both the average glide score of all reported poses (–6,2 in hDAT *vs.* –5,6 in hSERT) as well as the overall number of poses (103 in hDAT *vs.* 65 in hSERT) point towards favourable binding of the compounds to hDAT and thus emphasize hDAT over hSERT selectivity. Furthermore, in cluster 1 of hDAT, H-bonds are formed between the cationic nitrogen of the ligand and the protein, which is not the case in hSERT. However, no clear rationale for hDAT over hSERT selectivity with respect to the role of the C_α_ substituent could be derived. The majority of poses show the aromatic moiety of cathinones with a bulkier C_α_-substituent preferentially located in the B-site, with the C_α_ substituent pointing towards the center of the binding site. Nevertheless, a considerable amount of poses also have the aromatic moiety positioned in the C-site with the C_α_-substituent pointing towards Asp79, Val152, Tyr156 and Ser422 in hDAT and towards Asp98, Tyr176 and Ser438 in hSERT, where this leads to a considerable amount of clashes in both proteins.

Nevertheless, a considerable amount of poses also have the aromatic moiety positioned in the C-site with the C_α_ substituent pointing towards Asp79, Val152, Tyr156 and Ser422 in hDAT and towards Asp98, Tyr176 and Ser438 in hSERT, where this leads to a considerable amount of clashes in both proteins.

It is well known that subtle changes in ligand structure could lead to major reorientations in the binding mode, which recently has also been hypothesized for a series of 3,4-methylenedioxyamphetamine analogs and their binding to hDAT and hSERT.^18^ Briefly, two binding modes could be observed in docking studies. While the unsubstituted MDA and the *N*-methyl derivative MDMA preferentially showed one binding mode and the *N*,*N*,*N*,-trimethylammonium analog MDTMA exclusively exhibited a different binding mode, the *N*,*N*-dimethyl derivative MDDMA could alternate between the two binding modes. It is tempting to speculate that also in the case of the C_α_-substituted compounds two binding modes could simultaneously occur, with the one showing less steric clashes being preferred in hDAT.

### Molecular dynamics

In order to assess the stability of the docking poses obtained, 20 ns molecular dynamics simulations were performed for compound 3 (Fig. 9). As reflected by protein backbone and ligand RMSD plots over the whole simulation time (20 ns), all four complexes (Fig. 9) converged after approximately 10 nanoseconds of unrestrained simulation (with a maximum backbone fluctuation of 4.5 Å from the starting structure, File S7, ESI‡). In addition, the protein secondary structure element analysis reveals a perfect stability over the whole simulation time (File S7, ESI‡). The ligand remains in its initial binding pocket in all four simulations. In three of the simulations, the aromatic moiety stays within its initial subsite (B or C). In the hSERT simulation starting with the aromatic moiety in subsite C, a switch to subsite B after 12 ns was observed.

Overall, the simulations are showing more stable interactions with the ligand in hDAT than in hSERT (File S7, ESI‡). Phe76 in hDAT and Tyr95 in hSERT, which are located at equivalent positions in both proteins (Fig. 8), are showing interactions with the cationic nitrogen of the ligand, providing further justification for the restraint in the docking study. Stable pi–pi stacking interactions of the aromatic moiety with Tyr156 in hDAT and Tyr176 in hSERT (Fig. 9) can be observed in both proteins, therefore supporting the preference of the aromatic moiety being located in subsite B. This observation is in line with a more pronounced prevalence of cluster 1 in the docking study.

Taken together, the findings from molecular modelling studies support the experimentally observed selectivity of selected cathinones for hDAT over hSERT.

### Summary and conclusions

Integrated open data sources combined with workflow tools such as KNIME or Pipeline Pilot are very powerful for conducting complex queries in order to create consistent data sets for further analysis. However, postprocessing of the data by *e.g.* scaffold clustering using the popular method of Bemis–Murcko requires careful analysis and the expertise of a medicinal chemist. As the Bemis–Murcko scaffolds are based on rings connected by linkers, common substructures which define a certain SAR series might be split over different Murcko scaffolds. In our cathinone use case, the analogues were spread over four different scaffolds (identified by an alternative substructure search). Combining them allows creation of a data set of 56 compounds, the largest one analysed so far. SAR and docking studies as well as molecular dynamics simulations point towards a significant influence of the substituent at the C_α_-atom to the carbonyl group on the hDAT activity as well as on hDAT over hSERT selectivity.

The workflow used in this study to retrieve and process the data can be adapted to other protein targets and use cases. Additionally, it could be expanded in order to reflect the selectivity profile for a whole protein family or include off-targets of interest, for example. The two workflows (with and without hERG annotations included) are freely available from myExperiment (http://www.myexperiment.org/).

In the search for congeneric SAR series, we advise combining scaffold-based clustering methods with similarity searches (*e.g.* a common substructure search). Handling the processed data with caution, the methodology provides a useful way of exploring data if common substructures of compound series are less/not defined.

## Methods

### Workflow for data collection and data mining

Bioactivity data for the human serotonin transporter, dopamine transporter, and hERG potassium channel was collected from the Open PHACTS Discovery Platform by using its convenient API (version 1.5) in conjunction with specialized OPS-KNIME nodes (version 1.1.0).^47^ All further data filtering, preprocessing, and analyses were done in a single KNIME (version 2.11) workflow, which is fully flexible to be adopted to other protein targets and openly available from myExperiment (www.myexperiment.org).

The workflows consist of the following steps:

#### Retrieving pharmacology data from the open domain and endpoint filtering

The ‘Target Pharmacology: List’ API call was used to retrieve pharmacology data from ChEMBL_20^1^ for the protein targets under study by including a filter for the ‘activity_types’ (activity endpoints) ‘IC_50_’ and ‘*K*_i_’ as well as for the ‘activity_unit’ ‘nanomolar’. Upstream, input was given by providing the Uniform Resource Identifier (URIs) for the UniProt IDs of hSERT (P31645), hDAT (Q01959), and hERG (Q12809) in the form of a table. The pharmacology output was then preprocessed to exclude records with unspecified compound activity, and with activity values greater than 10^8^ (to avoid potential data errors). Further, activity values (for IC_50_ and *K*_i_ endpoints) were transformed into their negative logarithmic molar values (‘-logActivity values [molar]’). The same activity endpoints are available as ‘pCHEMBL values’ from the ChEMBL database, but in addition we also kept values with a relation different from ‘ = ’. Bioactivity values were also transferred into binary representation (active: 1, inactive: 0) by setting a cut-off value for separating actives from inactives. This cut-off was tailored to the specific target and bioactivity endpoint (*K*_i_/IC_50_): in the case of hSERT, sibutramine (*K*_i_ = 1.11 μM; IC_50_ = 2.09 μM), and in the case of hDAT, modafinil (*K*_i_ = 1.46 μM; IC_50_ = 1.83 μM) was selected as a reference and the listed bioactivities served as cut-offs. In the case of hERG we labelled all compounds with an IC_50_ or *K*_i_ below 10 μM as potential hERG blockers according to a study by Doddareddy *et al.* ^48^ In addition we inspected the ratio between the bioactivity for the primary target (hSERT or hDAT) and hERG for all drugs in the data set with measured hERG activity. Precise bioactivity values were always retained to be able to adjust the activity label(s) (0/1) in individual cases where bioactivities were close to the cutoff or due to a ‘>’ relation sign, which should be considered inactive although appearing active in our workflow.

#### Retrieving drug annotations

Annotations to known drugs and the preferred compound names of annotated drugs were retrieved from ChEMBL by utilizing the ‘ChEMBLdb Connector Input’ node in KNIME with input from the whole initial data set after preprocessing.

#### Splitting into two workflow strands

After data retrieval, filtering and preprocessing, two parallel workflow strands served for the extraction of a cathinone data set from a subset with IC_50_ endpoints as well as for a scaffold analysis on the whole data set (endpoints IC_50_ and *K*_i_). The filtering for the IC_50_ subset was done by a simple ‘Row Filter’. The subsequent ‘pivoting’ and filtering steps were done in parallel and independent for each of the workflow strands.

#### Creating overlap representations of pharmacology data and filtering

A pivot table was generated to display bioactivities of compounds against the two targets using the ‘Pivoting’ node in KNIME grouping rows by ChEMBL compound IDs and columns by ‘Target name’. If multiple activity values are given for the same compound–target pair, the median of those values was retained for the sake of visualization and classification, but preserving the list of all activity labels as well as the list of all precise bioactivity values assigned to a compound–target pair. Next, the data sets were filtered in order to keep only compounds with bioactivity measurements for both targets by using the ‘Numeric Row Splitter’ node.

#### Retrieving the cathinone data set

A substructure search for benzoylethanamine (= cathinone) was performed on the IC_50_ subset by using the ‘Substructure Search Node’ in the CDK module. Grouping by PubMed IDs served for getting informed about relevant literature sources.

#### Heat map representation

Starting from the larger data set with IC_50_ and *K*_i_ activity endpoints, compounds with contradictory activity classifications (if median activity labels of compounds = 0.5 for one/both of the transporters) have been removed for the sake of visualization. The resulting heat maps were visualized with the ‘HeatMap (JFreeChart)’ node in KNIME.

#### Scaffold analyses

Bemis–Murcko scaffolds of the compounds were retrieved by the node ‘RDKit Find Murcko Scaffolds’. Subsequently, compounds were grouped by their scaffolds. For analyzing scaffolds in the cathinone subset all scaffolds with more than one member compound were kept. For the whole data set, scaffold clusters with at least 10 unique compounds were kept for further analyses. Next, scaffolds with a preferential activity for one of the two targets and those showing activity on both targets (by evaluating their mean activity labels) were identified.

#### Performing a substructure search for various antidepressants

A substructure search for common substructures of antidepressants as retrieved after Murcko analysis was performed for three different scaffolds, which appeared as hSERT selective and drug-containing in our workflow. The ‘Substructure Search Node’ in the CDK module was used in order to look for additional compounds with the defined common substructure in the whole hSERT/hDAT data set (with IC_50_ and *K*_i_ endpoints).

### SAR analysis

**Fig. 10:**
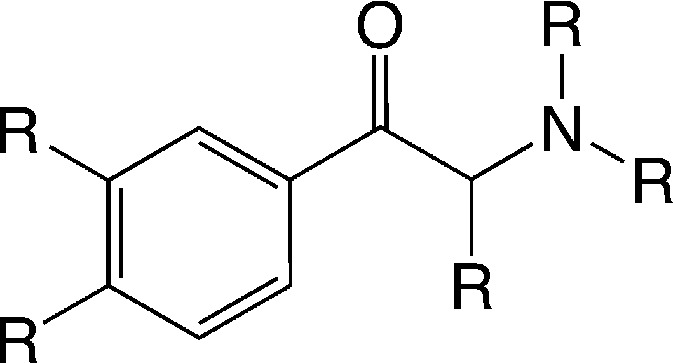
Common scaffold of the cathinones.

In order to get first insights into the molecular features triggering hDAT over hSERT selectivity of cathinones, a classical Hansch analysis using descriptors of lipophilicity, size, polarizability and electronic properties was performed. Van der Waals volume (vdw-vol), partition coefficient (log *P* (o/w)), and the molar refractivity of the compounds were calculated in MOE (molecular operating environment).^49^ The sigma Hammett constant was used as electronic parameter, and the respective values were picked from a table.^50^ To retrieve physicochemical parameters such as vdw-vol, log *P* (o/w), molar refractivity (mr), for individual substituents, we implemented an incremental approach using MOE. Briefly, the difference of the vdw-vol of two molecules which differ only in one position, *e.g.* a *para*-substituent on the aromatic ring, was used to calculate the incremental vdw-vol of this substituent. This allowed generation of a data matrix of substituent constants for all R-groups outlined in Fig. 10. Finally, we added two indicator variables displaying the presence or absence of a meta or para substituent on the aromatic ring. The SAR analysis was conducted in StatPlus for MAC using the linear regression function.

### Molecular docking study

For docking of a selected set of compounds the software package Glide 6.8 was used.^51^ Protein homology models for hSERT and hDAT in the outward facing conformation were taken from Saha *et al.*, 2015.^15^ As both visual inspection of the data as well as QSAR studies revealed an influence of the substituent at the alpha position to the carbonyl group, six compounds (compound **1–6**, Fig. 7) reflecting variations in this position (methyl-, ethyl-, propyl- and isobutyl-residues) were used for docking. Compounds were used in their protonated form reflecting their interactions in the binding pocket^45,52,53^ and in S-configuration due to higher activity reported.^17^ The proteins were prepared with Schrödinger Suite 2015-3 Protein Preparation Wizard; Epik version 3.3^54^ and in hDAT a water molecule was removed from the binding site. The center of the receptor grid is nortriptyline, which is co-crystallized in the template (PDB ; 4M48) for hDAT and paroxetine because of the higher affinity in hSERT. The template for the hSERT model is also PDB ; 4M48 due to its higher resolution, but the paroxetine ligand was used from PDB ; 4MM4, placed into the model by a structural alignment of the C_α_-atoms, and the resulting complex was protonated and energy minimized in MOE.^49^ Furthermore, as the antidepressant ligands in the crystal structures^45^ show an analogy in the positive partial charge density of the cationic nitrogen, in the cathinones it was forced to be placed within 2–4 Å to the backbone of the carbonyl oxygen of Phe76 in hDAT and Tyr95 in hSERT.^15^ For the output, the number of poses was limited to 100 per ligand.

To analyse the results, the poses were clustered with the support of two in-house scripts: the RMSD matrix of the common scaffold was calculated with a MOE script^49^ and the clusters with an R script at a defined maximal distance of 3 Å within one cluster.^55^ The underlying algorithm is a hierarchical clustering on a set of dissimilarities and techniques to analyse it.

### Molecular dynamics simulations

The MD study was performed by using the Schrödinger software with the Maestro suite (version 10.2^56^) for visualization and Desmond (version 4.2^57^) for the MD simulation. The four complexes gained from the docking study were prepared with the Protein Preparation Wizard.^58^ The force field used was OPLS2005, SPC was chosen as the solvent model and POPC (300K) as the membrane model. The system was placed in a box (using periodic boundary conditions) and neutralized with counter ions at a salt concentration of 0.15 M. Energy minimization was accomplished using a hybrid method of the steepest descent and the limited memory Broyden–Fletcher–Goldfarb–Shanno (LBFGS) algorithms. The maximum number of iterations was set to 2000, the convergence threshold for the gradient in units to kcal mol^–1^ Å^–1^ to 1. The simulation was conducted for 20 ns in total with recording intervals of 1.2 ps for the energy and 4.8 ps for the trajectory. The relaxation of the system before the simulation was performed using the standard protocol (NVT ensemble with Brownian dynamics at 10K with short time steps and solute non-hydrogen atoms restrained, NVT ensemble using Berendsen thermostat, NPT ensemble using a Berendsen thermostat and a Berendsen barostat). To analyze the results, the simulation event analysis as well as the simulation interaction diagrams incorporated in Desmond were generated and evaluated.
