# Supplementary material for: From linked open data to molecular interaction: studying selectivity trends for ligands of the human serotonin and dopamine transporter
Source: Medchemcomm. 2016 Jul 22;7(9):1819–31. doi: 10.1039/c6md00207b (PMC5100691; doi:10.1039/c6md00207b)
Supplement: Supplementary file 1 [file MD-007-C6MD00207B-s001.pdf]

This journal is © The Royal Society of Chemistry 2016

Figure S1

24 promiscuous hSERT/hDAT scaffolds

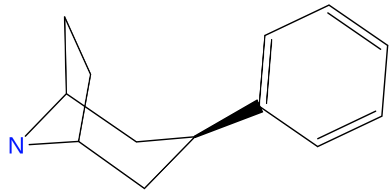

82

0.8780 0.9573

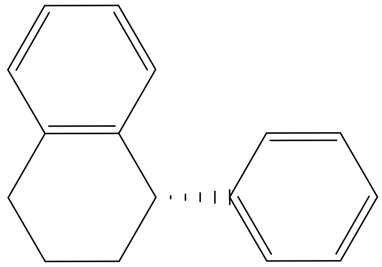

48

1.0000 0.8750

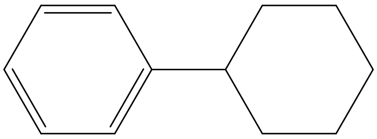

34

0.7059 0.7941

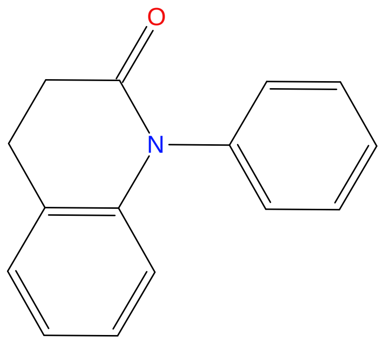

25

1.0000 1.0000

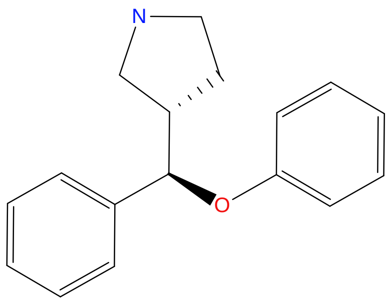

23

1.0000 0.9565

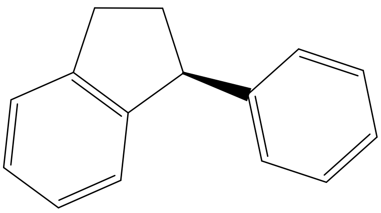

22

0.7500 0.9091

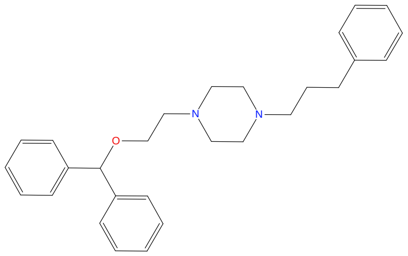

21

0.7143 1.0000

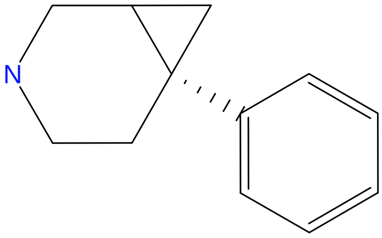

21

1.0000 0.8571

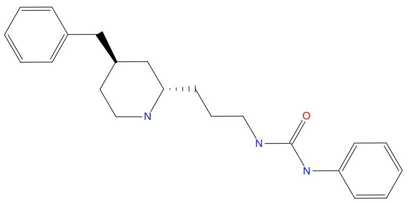

20

0.9500 0.8500

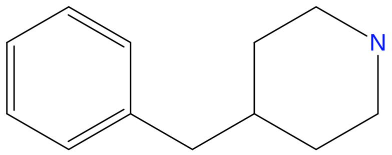

18

1.0000 0.8333

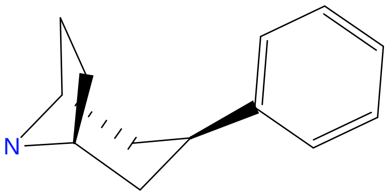

18

0.9444 1.0000

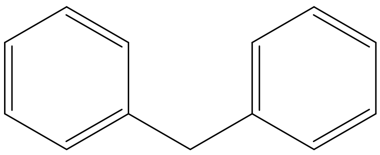

17

0.8235 0.7059

counts of unique compounds and mean activity labels (hSERT left, hDAT right)

Figure S1

24 promiscuous hSERT/hDAT scaffolds

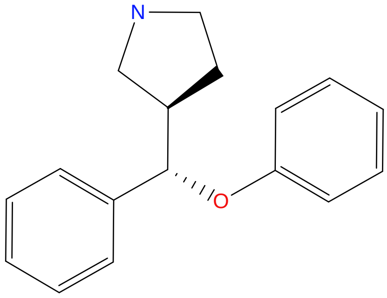

16

1.0000 1.0000

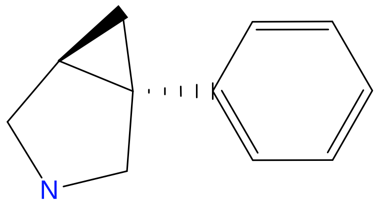

16

1.0000 1.0000

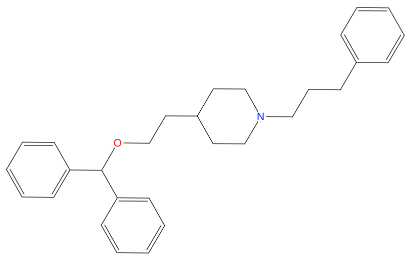

15

1.0000 1.0000

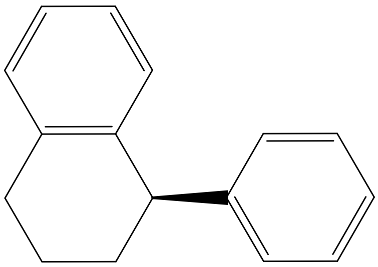

15

0.8667 1.0000

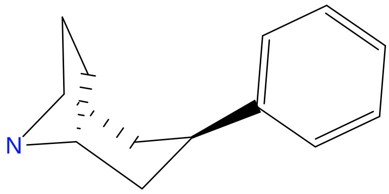

15

1.0000 1.0000

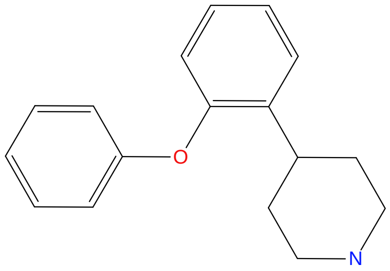

13

0.7692 0.9231

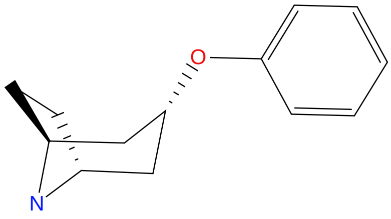

13

0.8462 0.6154

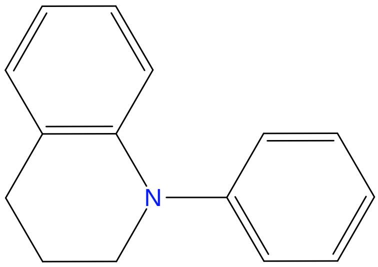

12

0.7500 0.9167

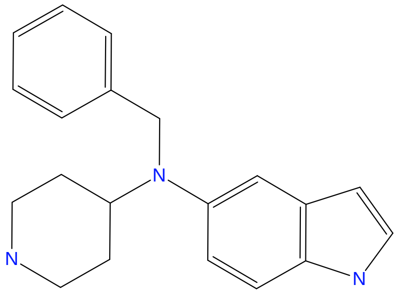

11

1.0000 0.9091

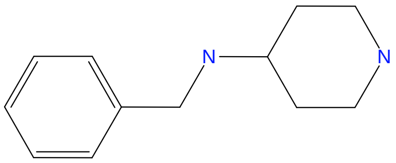

11

1.0000 1.0000

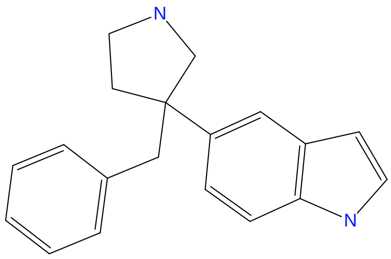

10

1.0000 1.0000

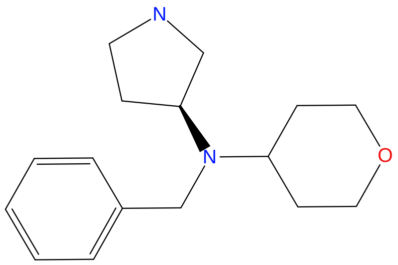

10

1.0000 0.7000

counts of unique compounds and mean activity labels (hSERT left, hDAT right)
