# Supplementary figures and images for: From linked open data to molecular interaction: studying selectivity trends for ligands of the human serotonin and dopamine transporter
Source: Medchemcomm. 2016 Jul 22;7(9):1819–31. doi: 10.1039/c6md00207b (PMC5100691; doi:10.1039/c6md00207b)

regarding publications of Imiquimod in literature (1980-2016)

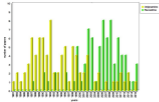

Supplement: Supplementary file 3 [file MD-007-C6MD00207B-s003.pdf]

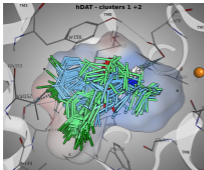

Supplement: Supplementary file 4 [file MD-007-C6MD00207B-s004.pdf]

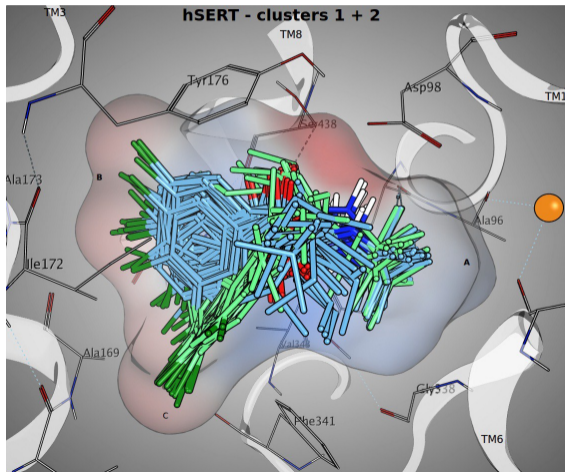

Supplement: Supplementary file 5 [file MD-007-C6MD00207B-s005.pdf]
