# Supplementary material for: From linked open data to molecular interaction: studying selectivity trends for ligands of the human serotonin and dopamine transporter
Source: Medchemcomm. 2016 Jul 22;7(9):1819–31. doi: 10.1039/c6md00207b (PMC5100691; doi:10.1039/c6md00207b)

Electronic Supplementary Material (ESI) for MedChemComm.
This journal is © The Royal Society of Chemistry 2016

Protein-Ligand RMSD

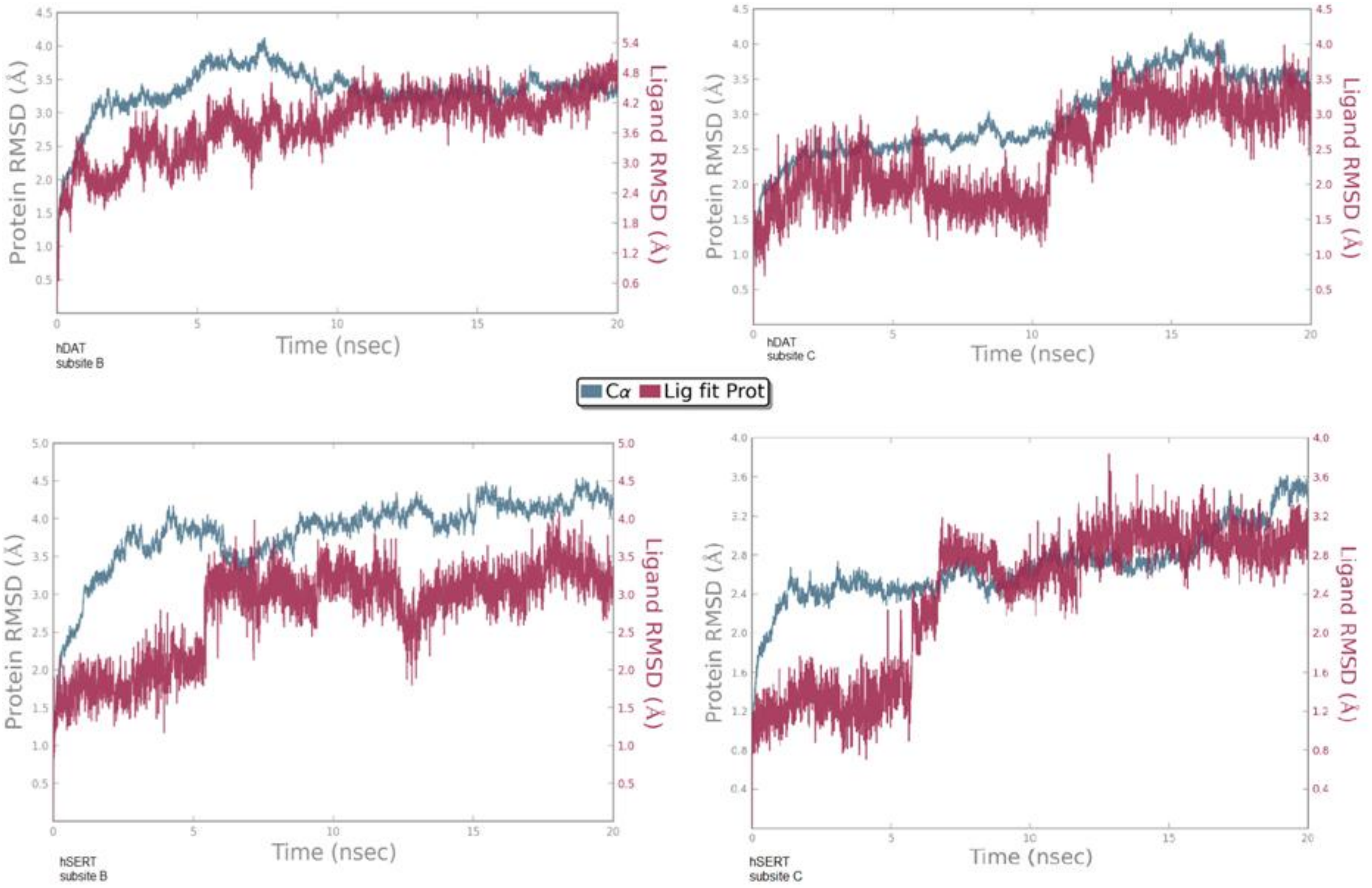

Protein Secondary Structure

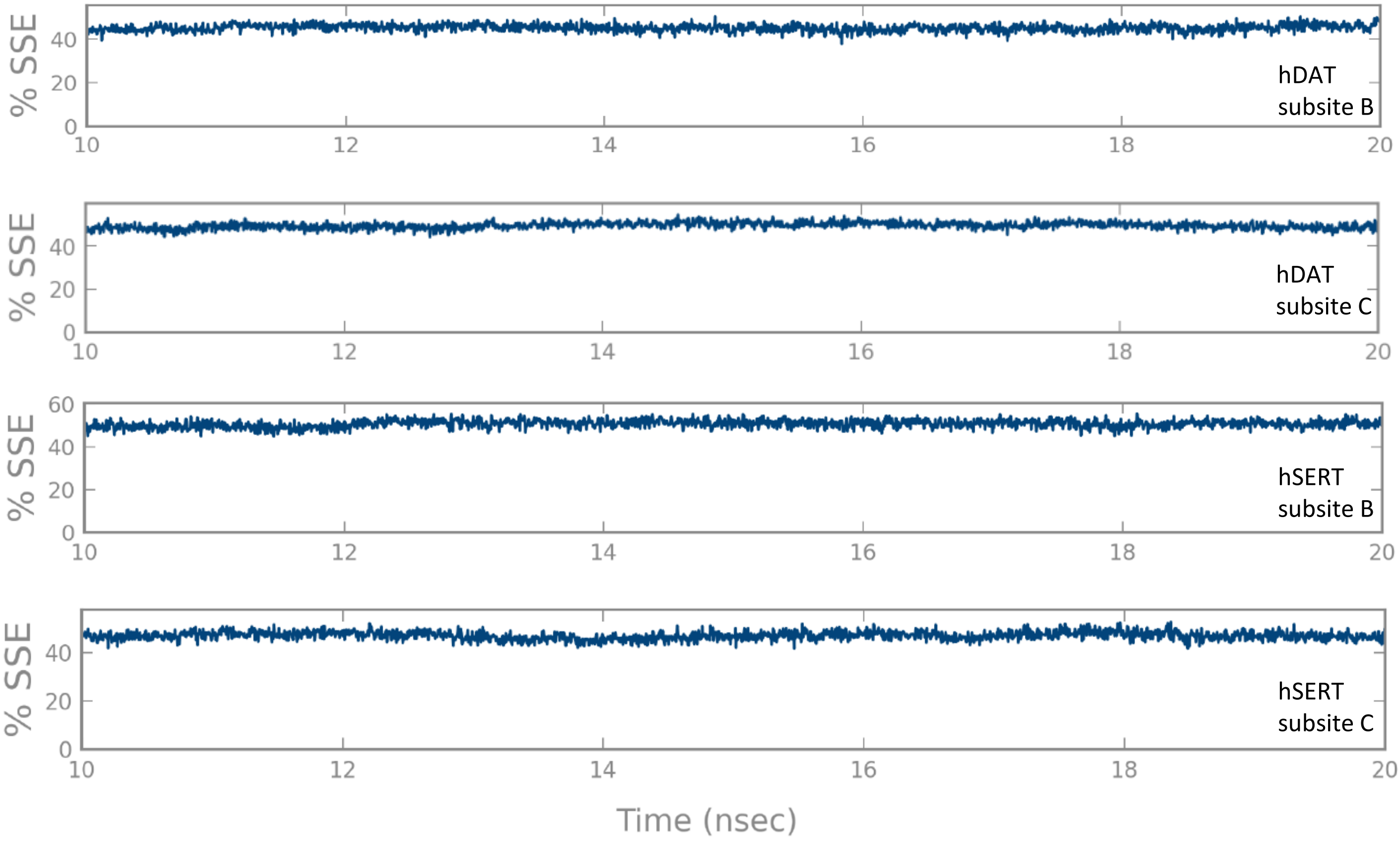

Protein-Ligand Contacts

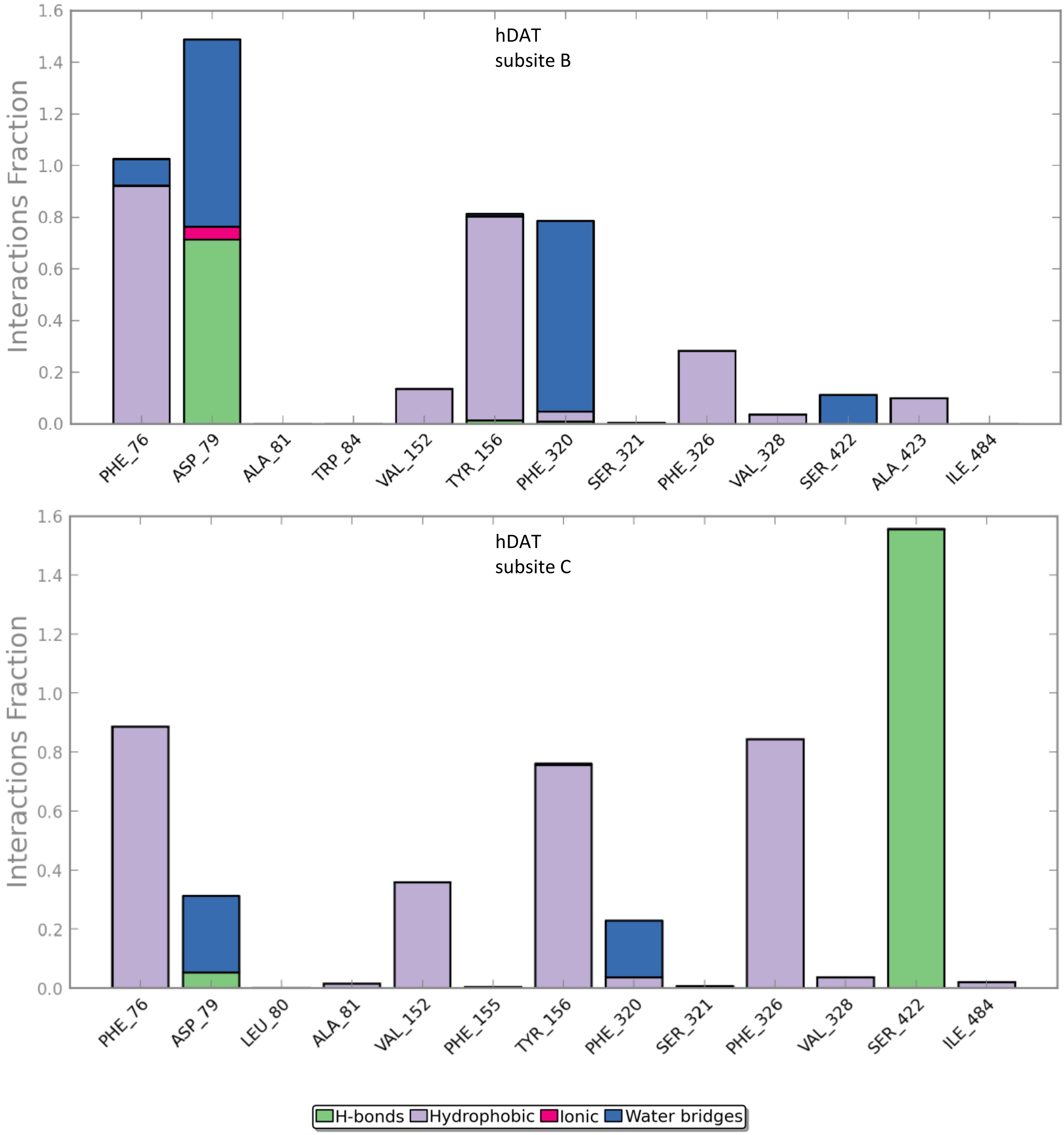

Protein-Ligand Contacts

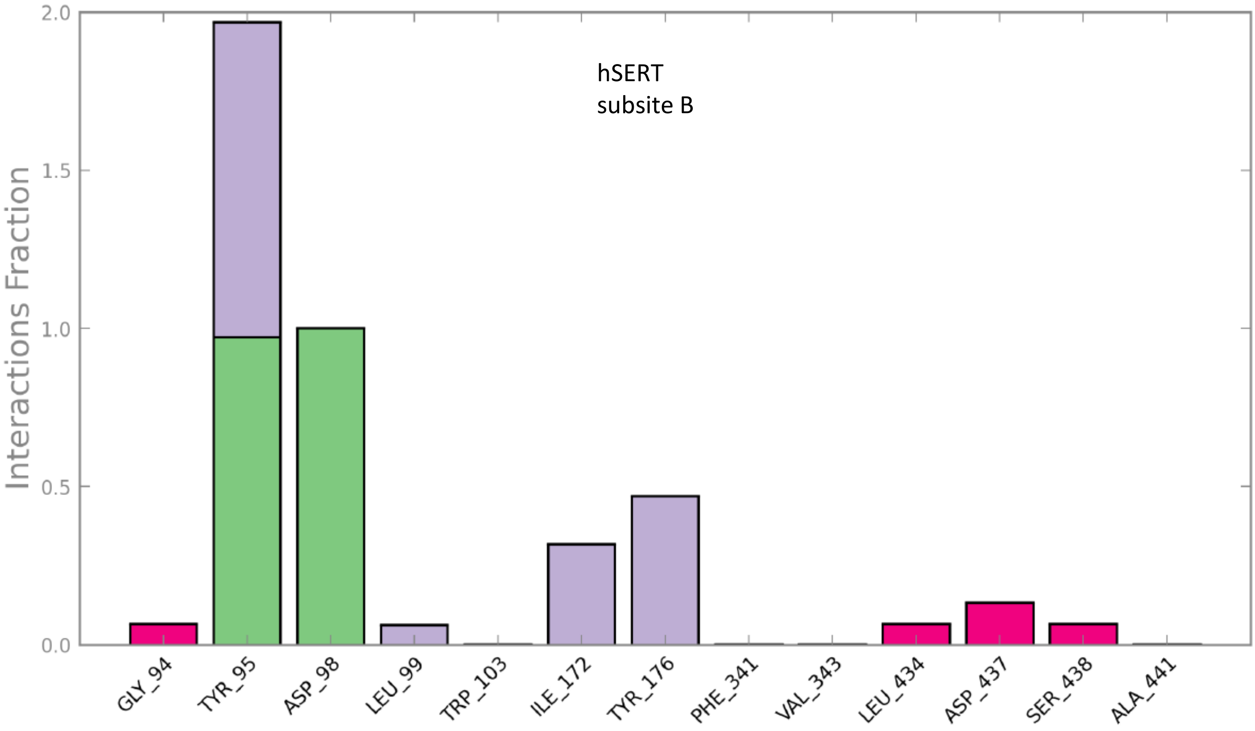

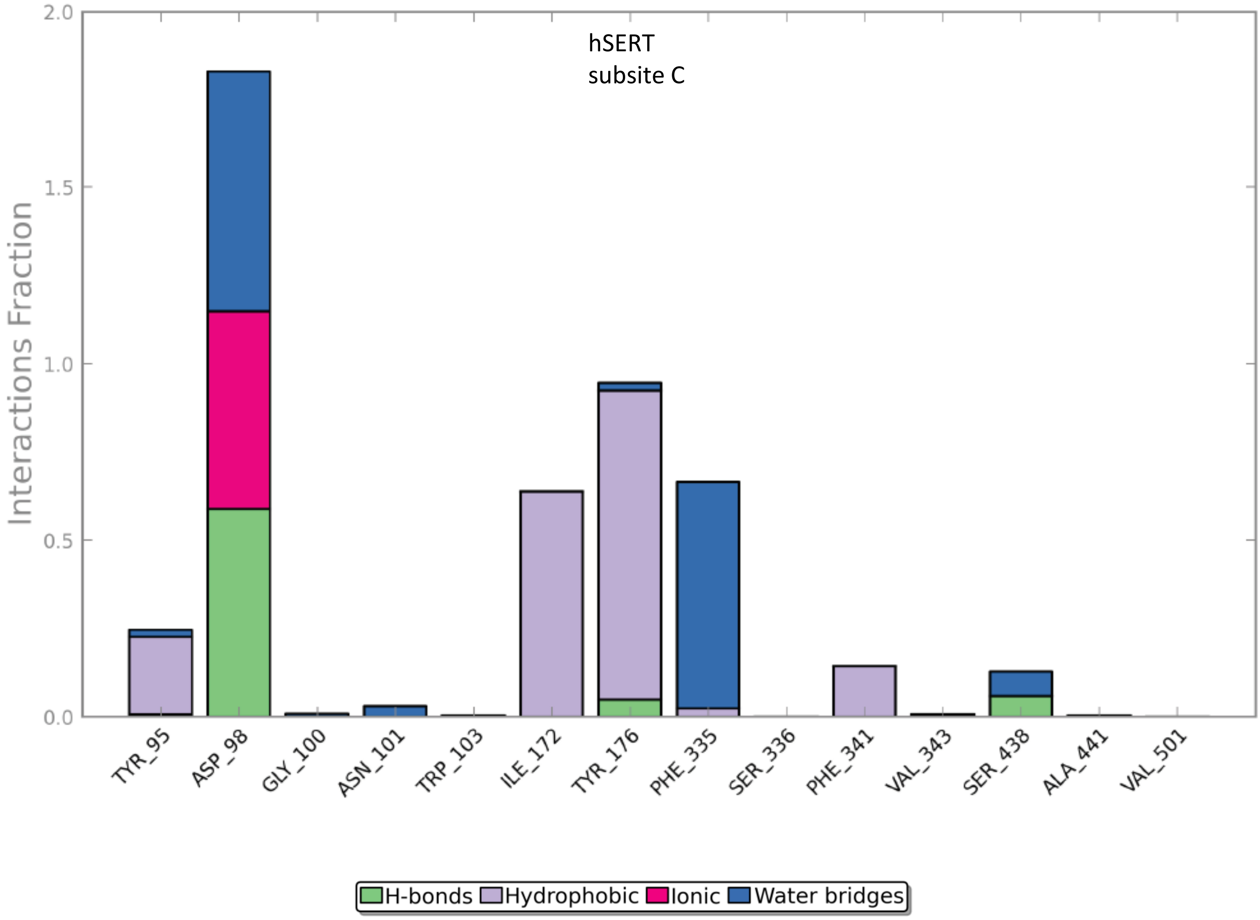

Protein-Ligand Contacts over time

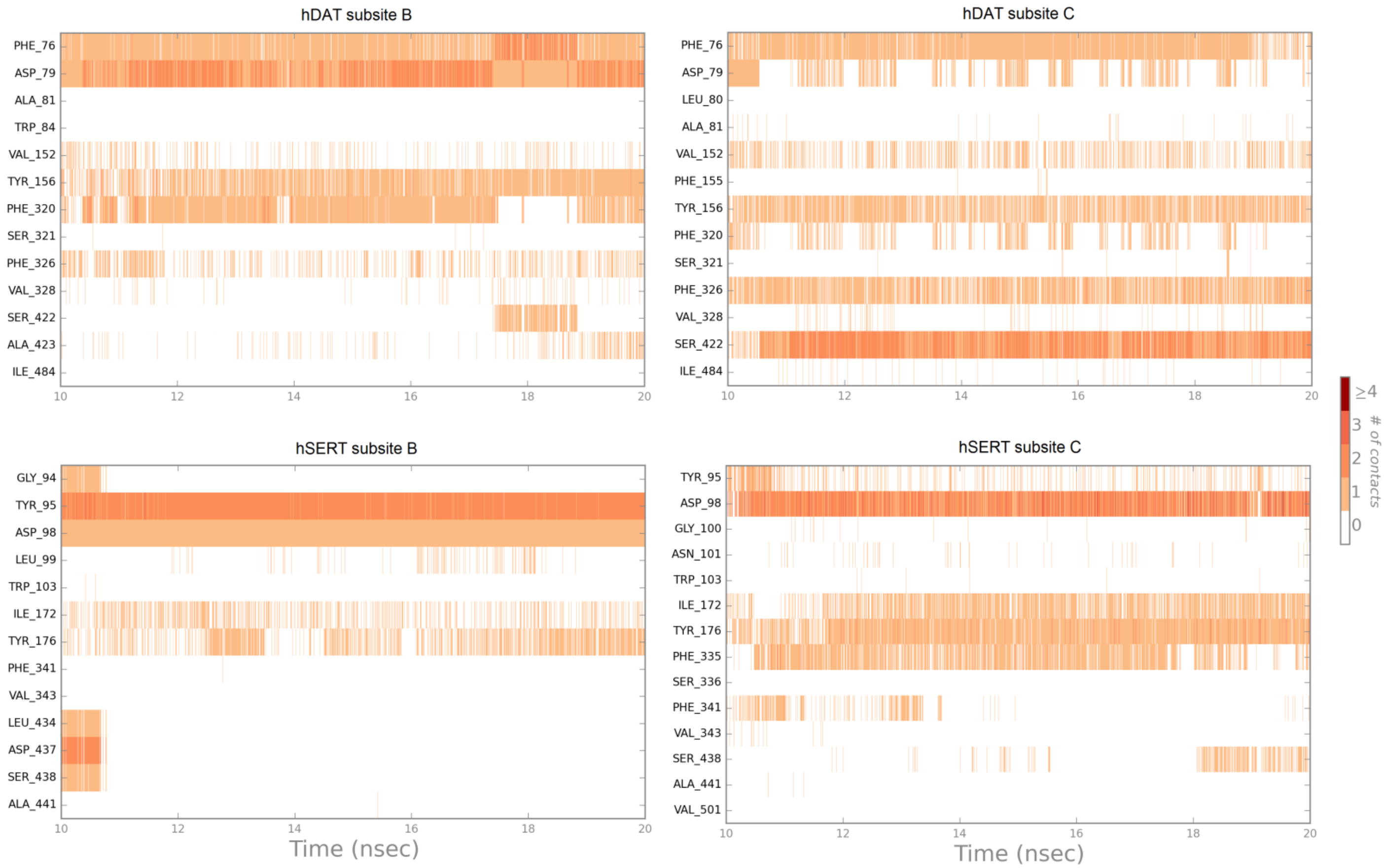

Supplement: Supplementary file 7 [file MD-007-C6MD00207B-s007.pdf]
